# Fractal Analysis of the Fracture Evolution of Freeze-Thaw Damage to Asphalt Concrete

**DOI:** 10.3390/ma12142288

**Published:** 2019-07-17

**Authors:** Jun Li, Fengchi Wang, Fu Yi, Jie Ma, Zhenhuan Lin

**Affiliations:** 1 School of Civil Engineering, Shenyang Jianzhu University, Shenyang 110168, China; 2 School of Transportation Engineering, Shenyang Jianzhu University, Shenyang 110168, China; 3 College of Architecture and Transportation, Liaoning Technical University, Fuxin 123000, China; 4 BIM (Building Information Modeling) Research Center, Shenyang Jianzhu University, Shenyang 110168, China

**Keywords:** freeze-thaw damage, computed tomography, fracture toughness, fractal dimension

## Abstract

AC (asphalt concrete)-13, as the main material used in pavement construction, has been applied widely in seasonal frozen areas. In order to understand the fracture mechanism in the freeze-thaw (F-T) damage process, the mesoscale structure of AC-13 is obtained by computed tomography (CT). The fractal dimension of cracks is used as a damage evaluation index. Most previous studies have only focused on the fractal dimensions of whole cracks, while ignoring the fractal tectonic process and the self-similarity degree of a single fracture. Therefore, in this study, the intrinsic mechanism of fractures and damage were investigated. In addition, the critical crack stress and fracture toughness models of a single fracture in a freeze-thaw damage process are established for AC-13. The results indicate that in terms of the critical crack stress and fracture toughness, with the increase of F-T times, there is an obvious decreasing trend. The fracture model can effectively describe the fracture toughness calculated by ABAQUS in the process of freeze-thaw cycles.

## 1. Introduction

Asphalt concrete is the most common pavement construction material for each level of roads; it is characterized by the advantages of energy savings, emissions reductions and superior skid resistance [1,2]. As we know, asphalt is both a thermoplastic and a cementing material, showing sensitivity to temperature changes. Nowadays, increased traffic loading and climate change have elevated the requirements for the asphalt concrete [3,4]. Especially in seasonal frozen areas, thermal cracking caused by the contraction and expansion of asphalt under freeze-thaw cycles causes major pavement damage, and could cause many disasters, such as pavement cracking and mud boiling [5,6,7]. These phenomena shorten the service life of asphalt concrete. Therefore, maintaining the stability of pavements in the process of freeze-thaw cycles has drawn more and more attention.

In seasonal regions, freeze-thaw damage is the most common damage for asphalt pavements, and the main cause of asphalt pavement disasters [8,9,10]. In the warm-thaw season, snow and ice melt into water and penetrate into the interior of asphalt pavement along cracks. In the winter-freeze season, the moisture in the surface layer does not discharge quickly and efficiently, and when the surface layer temperature is below 0 °C, the frost heave effect of water produces uneven stresses on asphalt pavements. In conclusion, with the process of moisture immersion and freeze-thaw cycles, the interface between aggregate and asphalt is weaken by frost heave stress, and the mechanical properties of the integral asphalt concrete gradually decrease [11]. In recent years, a number of researchers have focused their efforts on to the freeze-thaw damage mechanism of mechanical property degradation of asphalt concrete—and have made great progress. Miao et al. introduced entropy theory to describe the decay behavior of the three-dimensional macro and micro textures of asphalt surfaces. The results showed that entropy has significant advantages in describing the anti-skid performance of asphalt pavements in freeze-thaw cycles [12]. Xu, Gong and Geng et al. employed the information entropy theory, an X-ray CT scanner and digital image processing technology to identify the behavior of asphalt mixtures under freeze-thaw cycles, and explained the development process of F-T damage from a microscopic point of view [13,14,15]. Guo et al. investigated the deteriorating properties of NHSS modified asphalt under a freeze-thaw aging process, and found that such a process had a great impact on the thermal properties of NHSS modified asphalt [16]. Huang et al. established a three-dimensional failure criterion for asphalt mixtures after freeze-thaw cycles by triaxial tests in the laboratory. The results indicated that the multi-axial strength decayed significantly after 20 freeze-thaw cycles [17]. Wang et al. conducted a freeze-thaw split to evaluate the flexural resistance damage caused by freeze-thaw cycles [6,15,18,19,20]. 

The interface between the asphalt binder and mineral aggregate affected by freeze-thaw cycles determines the service life of asphalt pavement, because degradation and stress concentrations occur more easily in the interface [21]. When the is temperature below zero, the interface fracture propagation caused by frost-heave force is the main reason for freeze-thaw damage. Many researchers have attempted to explain the relationship between fracture properties and asphalt pavement cracking. Zhao et al. presented a new analysis method, including both dimensional and J-integral analyses based on classic fracture theory, in order to evaluate the fracture and fatigue properties of asphalt binder [22]. Omranian et al. compared the maximum stresses at failure, fracture toughness, and fracture energy by a semicircular asphalt concrete bending test; the velocity of fracture initiation, velocity of crack growth, and fragility index were proposed to better understand the fracture behavior of asphalt mixtures with respect to the mixtures’ crack resistance and its propagation [23]. Le et al. used a discrete element model to simulate the fracture behavior of asphalt mixtures at low temperatures; a series of BBR and SCB tests were undertaken in order to verified the model [24]. Doll et al. conducted fracture tests on semi-circular bend edge cracked specimens, and the fractures was recorded with a camera to allow digital image correlation (DIC) measurements [25,26] to be undertaken. Onifade proposed a hierarchical approach to evaluate fatigue cracking in asphalt concrete pavements, providing a systematic approach to understanding the fundamental mechanisms of pavement deterioration [27]. Liu et al. applied a cohesive zone model in the software ABAQUS to analyze crack propagation in asphalt layers to predict service life under cyclic loads with an initial onset macro-crack [28]. Xin et al. put forward a method to determine the material composition of small particle-size (SPS) asphalt mixtures to control cracks in asphalt pavements in order to improve effectively crack resistance [29]. 

While the viewpoint that crack propagation is the cause of damage to asphalt concrete materials has reached a consensus, most studies on the subject have only adopted fractal dimensions to describe the irregularities and self-similarities of the whole cracks, neglecting the fractal construction degree of a single crack. In this study, the models of critical crack stress and fracture toughness for a single fracture based on fractal characteristics will be established to better understand the intrinsic mechanisms of fractures and damage in the process of freeze-thaw cycles.

## 2. Experiment

### 2.1. Materials and Methodology

First, 90# petroleum asphalt was used as a cementing agent; the basic tested index is shown in Table 1. All the above basic indicators for asphalt meet the requirements of 90# road asphalt as specified in the “Technical Specification for Construction of Highway Asphalt Pavement” (JTG F40-2004) [30]. 

Coarse aggregate, fine aggregate, and mineral powder: both coarse and fine aggregates are basalts, and their density measurement methods are the basket and volumetric bottle methods, respectively. The quality test results of the mineral powder are shown in Table 2.

**Figure 1:**
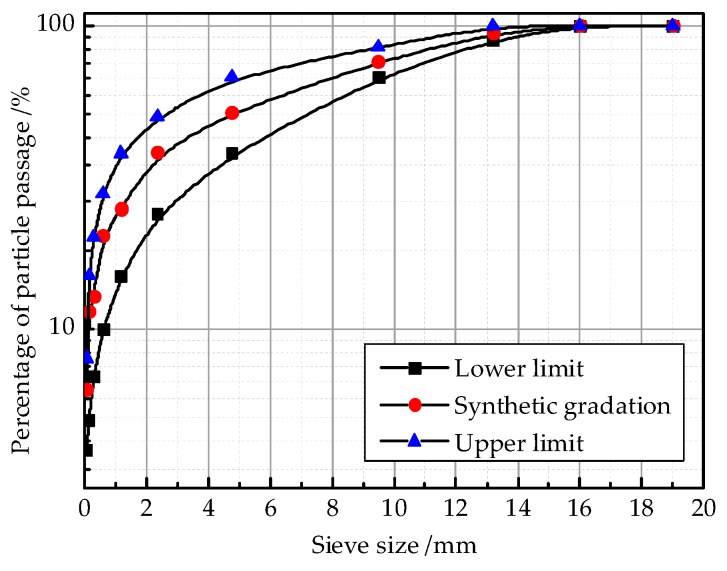
Aggregate gradation curve of AC-13.

In this study, asphalt concrete was used to study the fracture mechanical properties in the process of freeze-thaw cycles; the gradation curve of AC-13 is shown in Figure 1. The lower and upper limits were determined according to reference [30]. 

The ratio of the mixing mass of asphalt, sand, and powder was determined by a laboratory test. When the asphalt was melted and dehydrated, the specified sand and powder were stirred evenly. After that, the hot asphalt was poured into the preheated sand and powder based on the mixture amount, and then stirred evenly. Standard AC-13 specimens asphalt mortar with diameters and heights of 50 mm were made using a ZMJ-II automatic Marshall compactor. 

A freeze-thaw cycle test was carried out according to the standard testing methods, ASTM C666 and JTJ 270 [31]. One freeze-thaw cycle lasts about four hours and the core temperature of the asphalt concrete ranges from +8 ±2 Celsius to −17 ± 2 Celsius in one freeze-thaw cycle. When the freeze-thaw cycles reach 0, 1, 3, 6, 10 and 15 times, the asphalt concrete specimens were taken out of the equipment and CT gray images were obtained to evaluate the induced freeze-thaw damage.

### 2.2. CT Scanning

**Figure 2:**
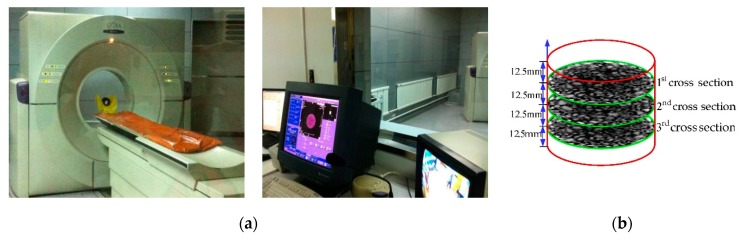
Digital image processing technology. (**a**) The Philips Brilliance CT scanner, (**b**) Determination of scanning layers.

**Figure 3:**
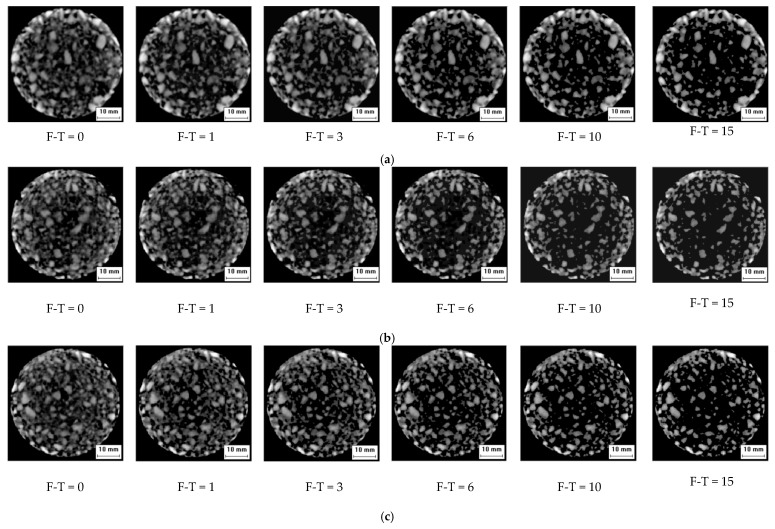
Digital image processing technology. (**a**) 1st cross section gray image, (**b**) 2nd cross section gray image, (**c**) 3rd cross section gray image.

Visualization is an emerging technology, that converts symbols obtained by optical equipment into 2D or 3D geometric shapes, and presents information in the form of specific images on a screen. Computed Tomography (CT), a type of visualization technology, was used to scan the internal structures of asphalt concrete specimens before and after freeze-thaw cycles. In this paper, the CT scanner used was manufactured by the Philips Brilliance center, Netherlands; scanning thicknesses ranged from 1mm to 15 mm (see Figure 2). The CT scanning gray images in the corresponding cross-section after freeze-thaw cycles are shown in Figure 3.

## 3. Results Analysis

### 3.1. Digital Image Processing Technology

**Figure 4:**
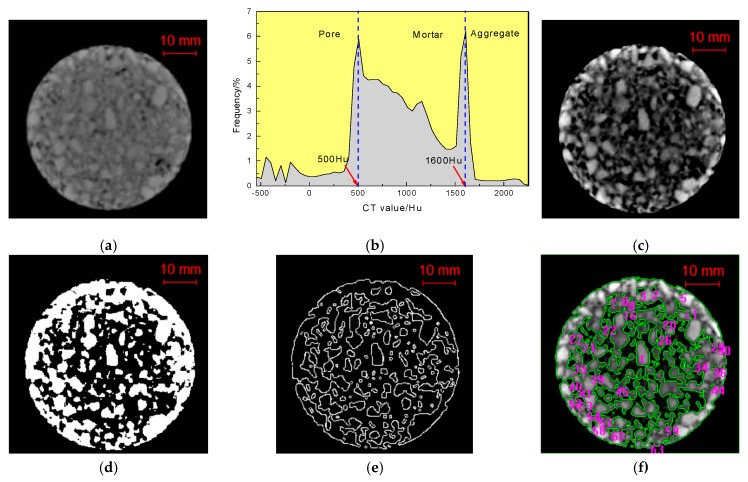
Digital image processing technology for the crack extraction procedure. (**a**) CT gray image, (**b**) CT number distribution, (**c**) Enhanced image, (**d**) Binary image, (**e**) Skeleton on binary image, (**f**) “AOI” selection.

Digital image processing (DIP) is a technology that removes noise, and enhances, restores, and segments. It also extracts features from images by software. In this paper, MATLAB 2016, IPP 6.0 (Image process plus) and MATHEMATICA are introduced to process and analyze the CT gray images. As shown in Figure 4, according to different CT value of asphalt concrete components, the CT number threshold was determined by the peak points of the CT number distribution curve in Figure 4b; this process is called threshold segmentation [32]. After enhancement of the original gray image in MATLAB, the boundary discrimination of each medium becomes more obvious. MATHEMATICA was used to extract the boundaries of the aggregate. 

IPP is used to analyze and calculate the geometric parameters of the processed DIP images. The crack boundaries are selected as the AOI (area of interest) to calculate the geometric parameters by the measurement function of IPP. The function of defining the scale based on the real size of the sample makes it possible to calculate and analyze accurately. 

**Figure 5:**
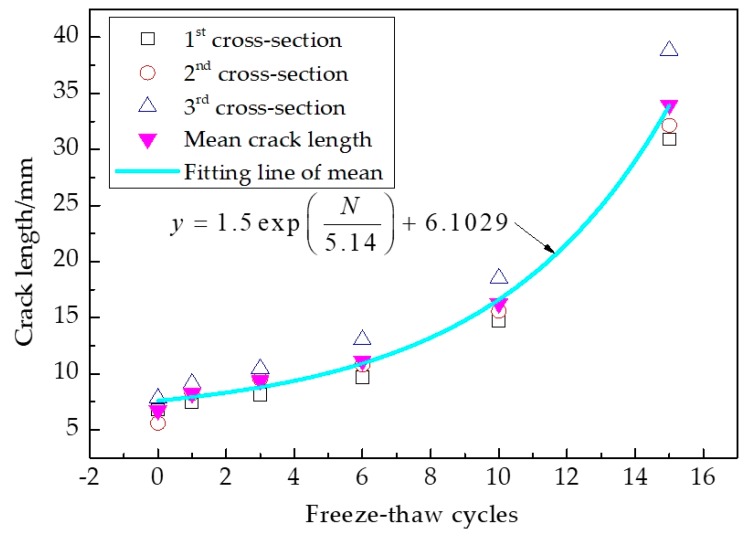
Relationship between crack length and freeze-thaw cycling.

From the binary image shown in Figure 4d, we can see that cracks after freeze-thaw cycling can be distinguished from each other, and the geometric parameters in such as area, i.e., perimeter, major axis length, and fractal dimension, can be easily calculated by the measurement function of IPP, which makes the quantitative analysis of pore meso-characteristics a reality. This CT scanner consists of three parts: a scanning system, the computer system, and the operating system. The thickness of the slices ranged from 1 mm to 10 mm, and the resolution was 0.5 mm × 0.5 mm. The scanning thickness was 2 mm, and the scanning interval was 3 mm in the experiment. As presented in Figure 5, the relationship between the crack length and the freeze-thaw cycles was established where the crack length is the sum of the crack lengths in each CT scanning section. The three cross-sections are selected as the analysis object, and the process of fracture analysis by IPP comprises importing the section pictures, defining scales, opening measurement functions, and exporting the data for analyzing. 

From Figure 5, the crack lengths increased with increasing freeze-thaw cycling. The results displayed that the crack length mainly increased after the peak point of the loading curve; the minimum and maximum values were 5.58 and 38.84 mm, respectively. From these results, crack lengths are found to be closely related to the damage degree of asphalt concrete. Cracks were always generated from pores and gradually propagated along with the aggregate and asphalt mortar interfaces. In addition, cracks branched and connected until the specimen failed. From Figure 3, the development paths of the cracks can be divided into two types: main and secondary cracks. The main cracks propagated along the interfaces of the aggregate and the asphalt mortar. The secondary cracks propagated around pores, and formed the branches of the main cracks.

### 3.2. 3D Reconstruction Technology

**Figure 6:**
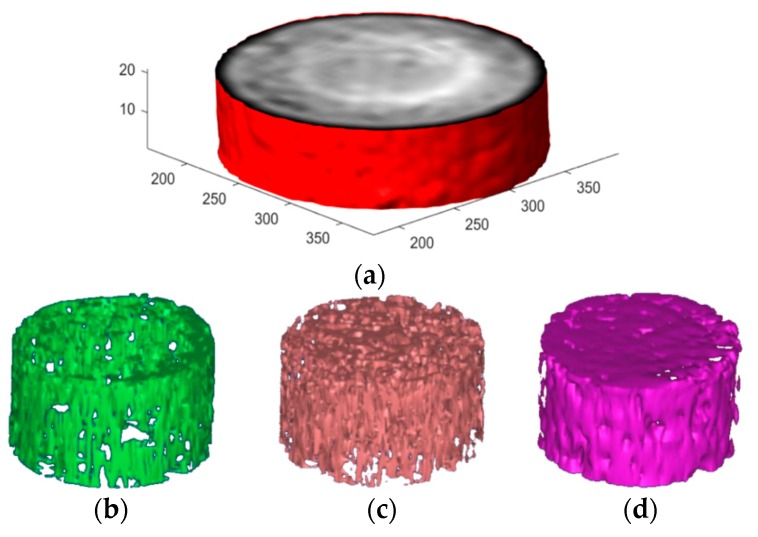
3D reconstruction and threshold segmentation. (**a**) 3D reconstruction effect, (**b**) Pore, (**c**) Asphalt mortar, (**d**) Aggregate.

The 3D internal structures in the spatial distribution of concrete aggregate, mortar, pores, and cracks were acquired using X-ray CT. Several 2D image slices of concrete specimens were put together and rendered to produce a 3D image. According to the law of three-phase threshold distribution of asphalt concrete (Figure 4b), the 3D distributions for aggregates, asphalt mortar, pores, and cracks are visualized via MIMICS, as shown in Figure 6. The reconstructed 3D image showed the characteristic of the spatial distribution of aggregates, asphalt mortar, and pores [33]. It was obvious that cracks from different angles merged as well as separated. The 3D reconstruction effect image and threshold segmentation images are shown in Figure 6a.

**Figure 7:**
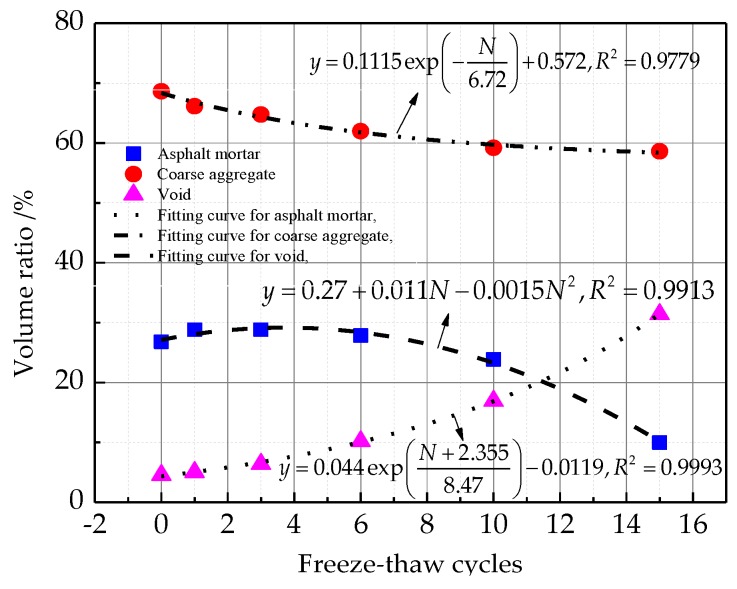
Three-dimensional reconstruction of components of AC-13 specimen.

As can be seen from the threshold segmentation images (Figure 6b–d), the three components of AC-13 asphalt concrete can be separated from each other by the segmentation threshold. The CT value of voids, asphalt mortar and coarse aggregate used in this paper were −450 Hu~500 Hu, 501 Hu~1600 Hu and 1601 Hu~2250 Hu, respectively. Coarse aggregate particles have similar contact characteristics. Asphalt mortar is closely distributed in the gap of the coarse aggregate and plays a good bonding role. The voids are closely distributed around the edges and sparsely distributed in the middle. This is characterized as “high around and low in the middle”, where it is related to the forming method of the specimen in the manufacturing process. The components of AC-13 specimens of the asphalt mixture not having undergone any freeze-thaw cycles were analyzed by micro-structure analysis. The change of volume ratio of each component is shown in Figure 7. 

As seen in Figure 7, with the increase of freeze-thaw cycling, the volume ratio of asphalt mortar goes up first, and then goes down. The volume ratio of coarse aggregate decreases all along the cycles, and the volume ratio of the void increases all along the cycles. This is mainly caused by the irreversible plastic volume deformation of the pore under the action of frost heaving force, and the loss of asphalt mortar along the crack channel with pore water [11]. As shown in Figure 7, the coefficients of correlations are all above 0.9779, indicating that the fitting equation explains the law on the change of volume ratio for each component reasonably.

### 3.3. Fractal Dimension

**Figure 8:**
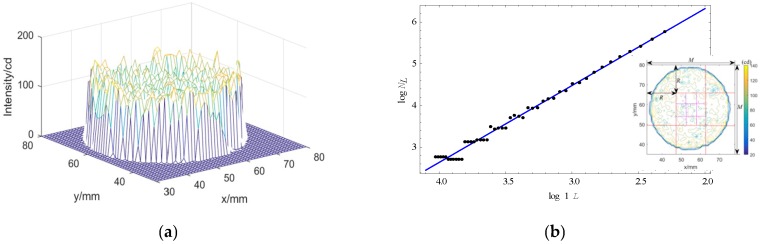
3D intensity distribution law and method to determine fractal dimension. (**a**) 3D intensity distribution, (**b**) Computing method of fractal dimension.

The fractal dimension reflects the validity of the space occupied by complex bodies, which is a measure of irregularity of complex shapes and bodies including the Hausdorff dimension, the box covering method, and so on. Consider a 3D space where two coordinates (*x*, *y*) represent a 2D position of each point in asphalt concrete, and the third coordinate (*z*) represents the optical intensity of image [34]; the 3D optical intensity is displayed in Figure 8a. The variation of intensity reflects the roughness of the surface and light absorptivity of the medium. As shown in Figure 8b, for a given image of size of *M* × *M*, we partitioned the 3D space into boxes of sides *L* × *L* × *L*′, where *L* is a given scale and is used as a multiple of the side length of a pixel in (*x*, *y*). *L*′ can be a multiple of the gray level unit in z direction. If *G* is the total gray levels, then *L*′ = *L* × *G*/*M*. Given a L×L grid at point (i,j), suppose that the minimum gray value is in box *b* and the maximum gray value is in box *u*, the minimum amount of the boxes that can cover the whole gray values in grid (i,j) is:

(1) $$n_{L}\left(i,j\right)=u-b+1$$

Then, the number of boxes that can cover all the patches can be calculated.

(2) $$N_{L}=\sum_{i,j}n_{L}\left(i,j\right)$$

Fractal dimension $d_{f}$ of the image is:

(3) $$d_{f}=\underset{L\to 0}{\lim}\frac{\log\left(N_{L}\right)}{\log\left(1/L\right)}$$

**Figure 9:**
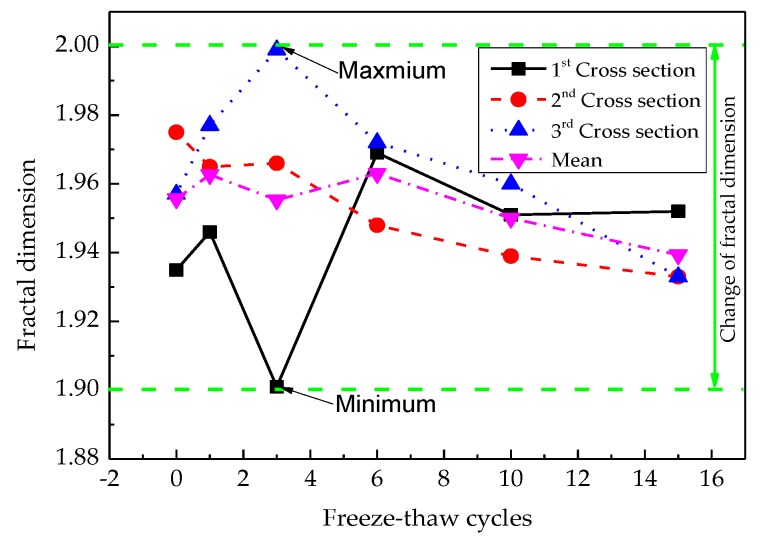
The law for the change of fractal dimension on each cross-section.

The fractal dimension on each section and the mean of all sections are shown in Figure 9. 

The mean fractal dimension of pore shows a fluctuation law at first, and then decreases gradually with the number of freeze-thaw cycles. It reaches the lowest value after the third freeze-thaw cycle and the highest value after the sixth freeze-thaw cycle. After more than 6 freeze-thaw cycles, the fractal dimension reaches a stable level, which indicates that the pore complexity at this time also reaches a stable level. This is mainly due to the joint action of pore expansion and pore closure. After more than six freeze-thaw cycles, the interaction between the two reached an approximate dynamic equilibrium, which is consistent with the results in reference [35]. 

### 3.4. Fractal Construction of Cracks

**Figure 10:**
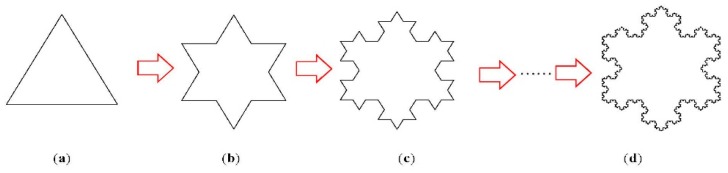
Koch snowflake construction process. (**a**) Initial element, (**b**) 1st construction, (**c**) 2nd construction, (**d**) 6th construction.

In order to simulate the fractal characteristics of fracture structures, the Koch curve is used. The Koch curve is a kind of typical fractal curve, which was first proposed by Koch, H.von in 1904 [36] and is now widely used to analyze specific and complex engineering problems. The construction process of the Koch fractal curve is shown in Figure 10. The more tortuous the external boundary of the fracture, the higher degree the fractal construction. The Koch snowflake, with a strong characteristic of self-similarity, can be used to simulate the characteristics of irregular edges for fracture propagation in the process of freeze-thaw cycles. 

As shown in Figure 10, the construction rule can be determined by the first step of the fractal construction, and by fractal construction size δ. After fractal construction, it can be expressed as [37]:

(4) $$\delta =a_{0}/3^{n}$$

where *n* is the number of constructions, and $a_{0}$ is the half projection length. 

**Figure 11:**
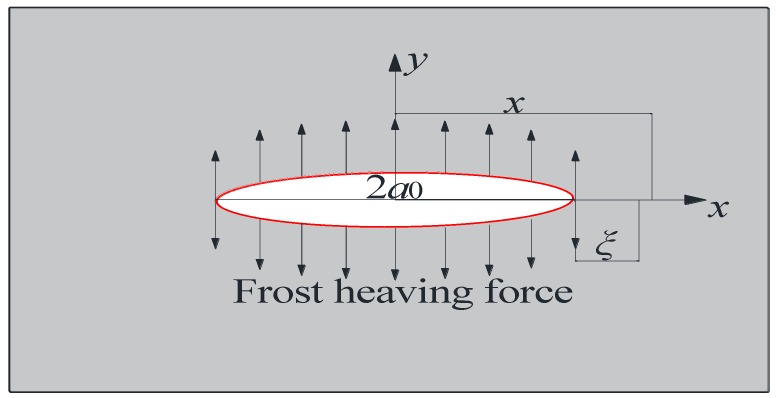
Type I fracture stress state.

Since the frost heaving force is the driving force of crack development, a mode I crack on the infinite plane plate was used to study the development of cracks during freeze-thaw cycling (Figure 11). The edge of cracks has the obvious characteristic of self-similarity caused by the effect of fractal construction. The relationship between the actual fractal length and the projection length can be expressed as follows [38]:

(5) $$a=a_{0}^{d_{f}}\delta^{1-d_{f}}$$

where $a_{0}$ is the projection length, a is the actual fracture length, and δ is the size of fractal structure. As the edge of crack is made up of two parts, the perimeter *p* can be determined.

(6) $$p=4a=4a_{0}^{d_{f}}\delta^{1-d_{f}}$$

If the crack is constructed using the Koch curve, then the perimeter of the crack is equal to the Koch fractal curve length of a certain construction number is which is taken as a criterion to determine the number of fractal constructions. According to Equations (4) and (6), the fractal construction number (*n*) can be expressed as:

(7) $$n=\frac{1}{\log3}\left[\log a_{0}-\frac{1}{1-d_{f}}\log\left(\frac{p}{4a_{0}^{d_{f}}}\right)\right]$$

**Figure 12:**
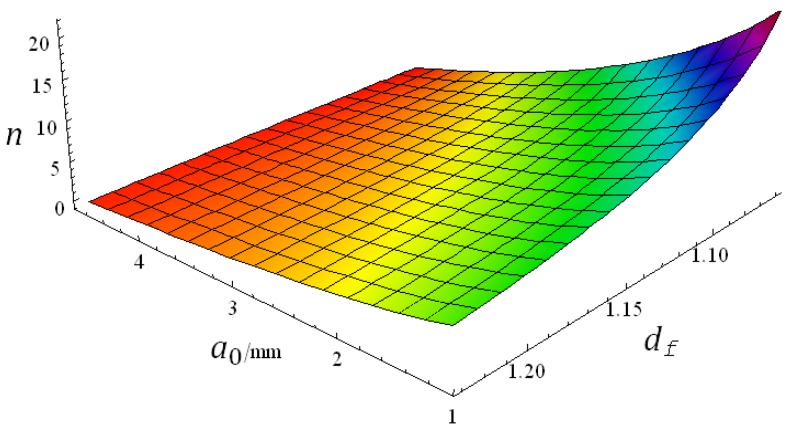
The relations among fracture construction times, fractal dimension, and fracture size.

For a certain crack perimeter, the number of fractal constructions is determined by $a_{0}$ and $d_{f}$ in Equation (4). MATHEMATICA is a mathematical computing software which combines numeric and symbolic computing functions. By programming Equation (7), complex mathematical relations can be visualized in 3-D. As shown in Figure 12, the 3-D visualization image is displayed. 

**Figure 13:**
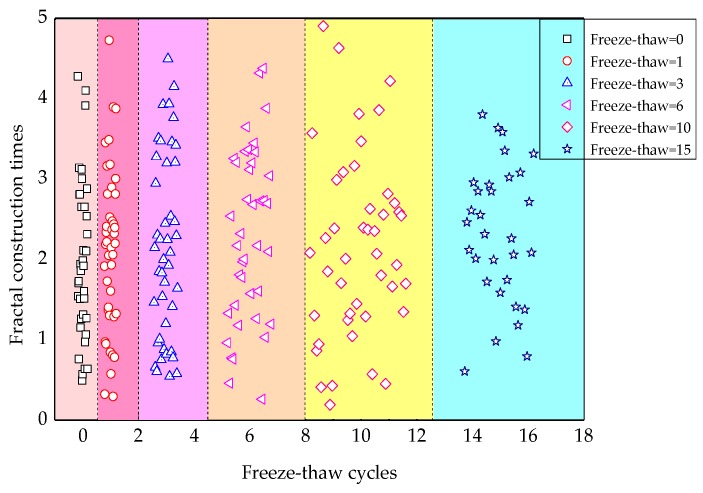
The change of fractal construction times with freeze-thaw cycles.

**Figure 14:**
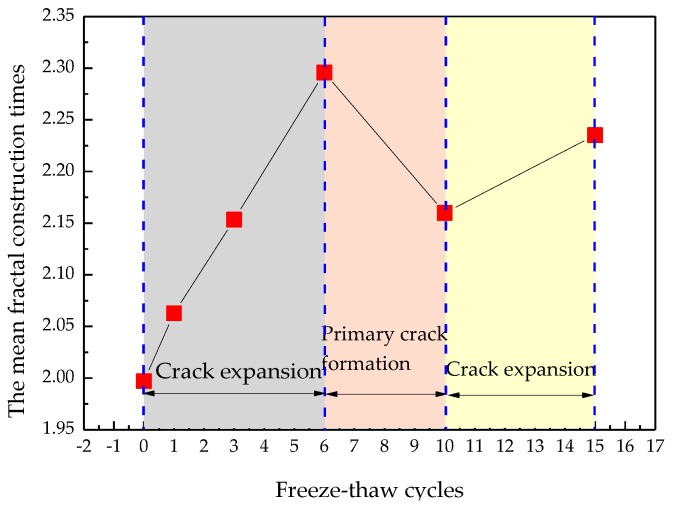
The relationship between the mean fractal construction times and freeze-thaw cycles.

Seen from Figure 12, when $a_{0}$ is small, n decreases with the increase of fractal dimension $d_{f}$, whereas when $a_{0}$ is large, the effect of $d_{f}$ on n is not obvious. The fractal construction times with freeze-thaw cycling of all cracks are shown in Figure 13. The mean of fractal construction times on the 1st cross-section are displayed in Figure 14.

As can be seen in Figure 13, the fractal construction times ranged from 0.19 to 4.9, and showed non-integer characteristics. Crack expansion can be considered as the process of Koch snowflake construction, but the degree of the fractal construction is relatively low. As seen from Figure 14, when fewer than freeze-thaw cycles were carried out, the construction times went up with increasing numbers of freeze-thaw cycles. When the number of freeze-thaw cycles ranged from 6 to 10, the construction times went down with increasing the number of freeze-thaw cycles. In contrast, when the number of freeze-thaw cycles was greater than 6, the mean construction times increased again. This is the result of an alternation of two functions of the original crack expansion and primary crack formation [22]. By this means, the fractal size δ of Equation (4) can be determined. 

## 4. Discussion

### 4.1. Fracture Toughness

Under the action of frost heaving force, the crack edge of asphalt concrete, as a heterogeneous material, has self-similar characteristics, which can be measured by the fractal dimension. For Euclidean two-dimensional space, the relationship between the perimeter (*p*) and sectional area (*A*) is $p\propto A^{1/2}$; for a circle, $p=2\sqrt{\pi }A^{1/2}$; for a square, $p=A^{1/2}/4$. Therefore, for a fractal space with fractal dimension of $d_{f}$, the relationship between the effective fractal perimeter (*p*) and section area (*A*) is $p^{1/d_{f}}\propto A^{1/2}$ [39]. Then, we can get:

(8) $$p^{1/d_{f}}=c\cdot A^{1/2}$$

**Figure 15:**
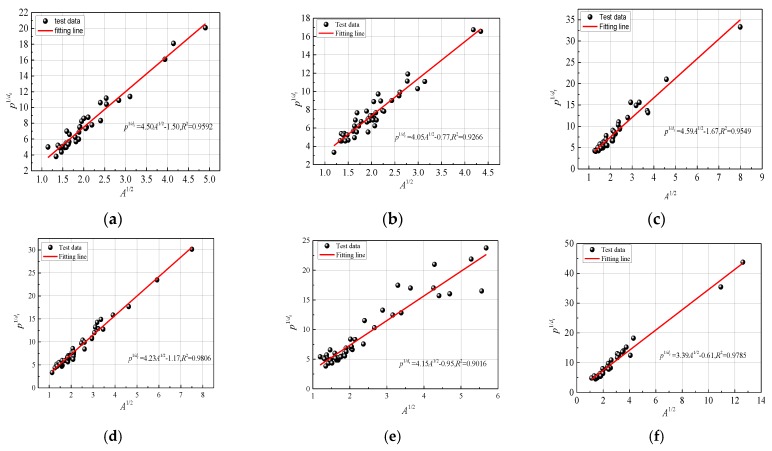
The Relationship between $p^{1/d_{f}}$ and $A^{1/2}$. (**a**) F-T = 0, (**b**) F-T = 1, (**c**) F-T = 3, (**d**) F-T = 6, (**e**) F-T = 10, (**f**) F-T = 15.

According to Equation (8), theoretically, there is a good linear relationship between $p^{1/d_{f}}$ and $A^{1/2}$. The coefficient of correlation $R^{2}$ can be used as a criterion for their linear correlation. For a certain number of freeze-thaw cycles, each crack boundary is isolated and closed. The fractal dimension will fluctuate in a small interval which is closely related to the damage caused by freeze-thaw cycles. The linear relationship between $p^{1/d_{f}}$ and $A^{1/2}$ can be fitted as:

(9) $$p^{1/d_{f}}=cA^{1/2}+m$$

where *c* is the slop of the fitting line, and *m* is the intercept of the fitting line. The fitting effects of the linear relationships are shown in Figure 15.

As shown in Figure 15, there is a strong linear correlation between $p^{1/d_{f}}$ and $A^{1/2}$; the correlation coefficients are all above 0.9, which indicates that the fracture evolution under freeze-thaw conditions has good fractal characteristics. Therefore, parameter *c* can be determined, as shown in Equation (12). The critical fracture stress can be calculated.

### 4.2. The Critical Cracking Stress

According to Equation (6), the surface free energy Π should be expressed as:

(10) $$\Pi =pt\gamma =4a_{0}^{d_{f}}\delta^{1-d_{f}}t\gamma$$

where t is the thickness of the plate, γ is the unit free energy density, J/m^2^. 

Based on Equations (6) and (10), the expression of fractal area A(δ) can be determined.

(11) $$A\left(\delta \right)=c^{-2}{\left(4a_{0}^{d_{f}}\delta^{1-d_{f}}\right)}^{2/d_{f}}=c^{-2}4^{2/d_{f}}a_{0}^{2}\delta^{\frac{2\left(1-d_{f}\right)}{d_{f}}}$$

When the minor axis of fractal crack approaches zero, the increment of strain energy ΔU is

(12) $$\Delta U=\frac{t\sigma^{2}A\left(\delta \right)}{2E}=\frac{t\sigma^{2}4^{2/d_{f}}a_{0}^{2}}{2c^{2}E}\delta^{\frac{2\left(1-d_{f}\right)}{d_{f}}}$$

where E is the modulus of elasticity, as determined by an indoor experiment. The symbol σ is the frost heaving stress, introduced by the freezing of water.

The process of crack growth is accompanied by the accumulation and transformation of energy. Therefore, it is reasonable to assume that the total potential energy increment is caused by frost heaving force, which is determined by the increment of strain energy ΔU and surface free energy Π. This can be calculated as follows:

(13) $$P=-\Delta U+\Pi =-\frac{t\sigma^{2}4^{2/d_{f}}a_{0}^{2}}{2c^{2}E}\delta^{\frac{2\left(1-d_{f}\right)}{d_{f}}}+4a_{0}^{d_{f}}\delta^{1-d_{f}}t\gamma$$

According to the potential energy extremum principle, when the released strain energy is exactly equal to the surface energy, the crack is in the critical equilibrium state. Meanwhile, the boundary conditions for the maximum of the total potential energy can be expressed as [40]:

(14) $$\frac{\partial P}{\partial a_{0}}=0,\frac{\partial^{2}P}{\partial a_{0}^{2}}<0$$

By introducing Equation (13) into Equation (14), we can obtain:

(15) $$-\frac{t\sigma^{2}4^{2/d_{f}}a_{0}}{c^{2}E}\delta^{\frac{2\left(1-d_{f}\right)}{d_{f}}}+4d_{f}a_{0}^{d_{f}-1}\delta^{1-d_{f}}t\gamma =0$$

Simplifying Equation (15), critical cracking stress $\sigma_{c}$ can be calculated as follows:

(16) $$\sigma_{c}=\frac{2c}{4^{1/d_{f}}}\sqrt{d_{f}a_{0}^{d_{f}-2}\gamma E\delta^{3-\frac{2}{d_{f}}-d_{f}}}$$

**Figure 16:**
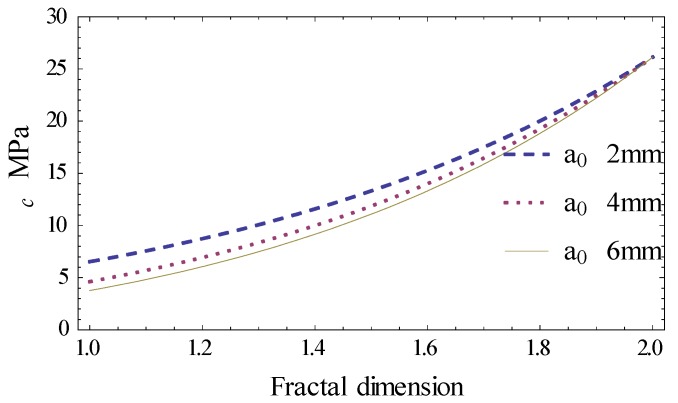
The relationship between $\sigma_{c}$ and $d_{f}$.

The relationship between critical cracking stress $\sigma_{c}$ and fractal dimension $d_{f}$ with different projection lengths of cracks is shown in Figure 16. So, the fracture parameters of AC-13 asphalt concrete can be determined and shown below:

The free surface energy *γ* = 0.5 J·m^−2^;
The fractal proportional coefficient is *c* = 4.05;
The fractal size *δ* = $a_{0}$/3^2^.

**Figure 17:**
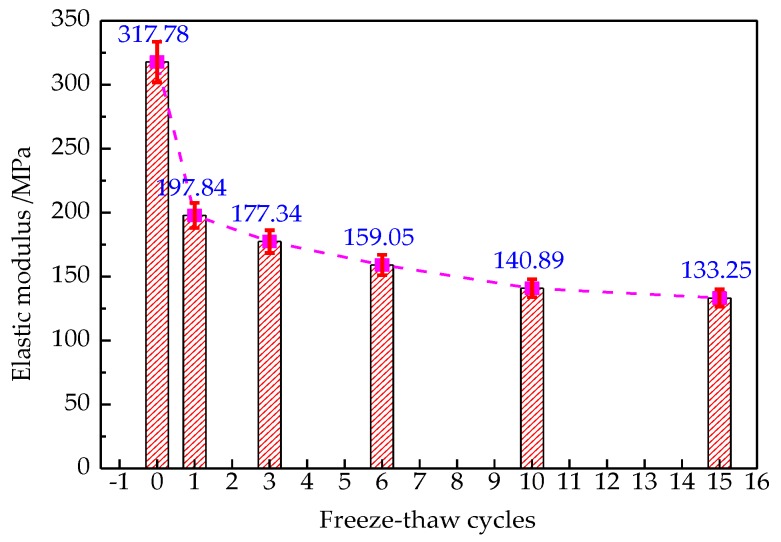
Change of elastic modulus.

**Figure 18:**
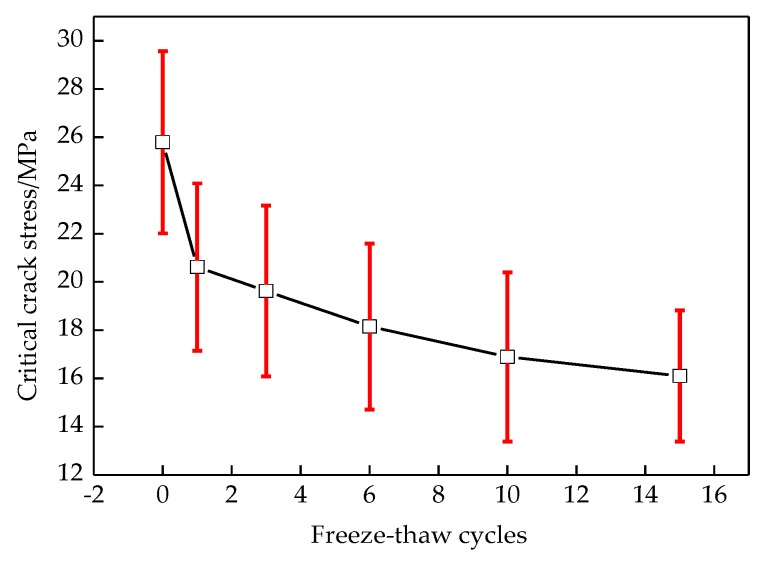
Change of critical crack length with freeze-thaw cycles.

The relationship between elastic modulus and critical crack stress is shown in Figure 17 and Figure 18, respectively. 

As seen in Figure 17, the elastic modulus deteriorates with increasing the freeze-thaw cycles, and decreases rapidly in the initial freeze-thaw cycles. This is due to the micro cracks in asphalt concrete caused by freeze-thaw cycle damage, which leads to a considerable decline in overall integrity and brittle fracture in advance. This is why many people define the degree of damage by modulus degradation [41]. As seen in Figure 18, the critical crack stress decreases with increasing the number of freeze-thaw cycles; this is because of the decline of the tensile strength at the interface due to freeze-thaw damage. 

### 4.3. Intensity Factor of Frost Heave Stress

**Figure 19:**
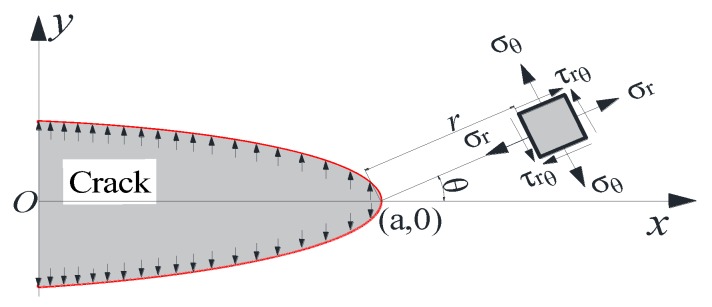
Stress field of frost heaving force at crack tip.

**Figure 20:**
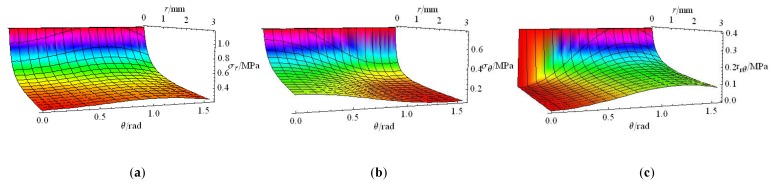
The relationship among stress field of $\sigma_{ij}$, *θ* and *r.* (**a**) $\sigma_{r}$, (**b**) $\sigma_{\theta }$, (**c**) $\tau_{r\theta }$.

Based on the theory of elastic fracture mechanics [42], the components of the stress field for the tip of cracks in Figure 19 can be determined using the following equations.

(17) $$\left\{ \begin{array}{l} \sigma_{r}=\frac{K_{I}^{f}}{\sqrt{2\pi r}}\cos\frac{\theta }{2}\left(1+\sin\frac{\theta }{2}\sin\frac{3\theta }{2}\right)\\ \sigma_{\theta }=\frac{K_{I}^{f}}{\sqrt{2\pi r}}\cos\frac{\theta }{2}\left(1-\sin\frac{\theta }{2}\sin\frac{3\theta }{2}\right)\\ \tau_{r\theta }=\frac{K_{I}^{f}}{\sqrt{2\pi r}}\cos\frac{\theta }{2}\sin\frac{\theta }{2}\sin\frac{3\theta }{2} \end{array}\right.$$

where $K_{I}^{f}$ is the stress intensity factor of Griffith I mode crack caused by frost heaving force, θ is the deflection angle of $\sigma_{r}$ relative to the major axis, and r is the distance between stress element and the crack tip. The change of each component with the variations of θ and r in Equation (17) is shown in Figure 20.

Based on basic fracture theory [43], $\sigma_{y}={\sigma_{\theta }|}_{\theta =0}$ is the driving force for cracking development. The boundary conditions are:

when *y* = 0, |x|<a, $\sigma_{y}=\tau_{xy}=0,\sigma_{y}=p$.
when |x|→∞, $\sigma_{x}=\sigma_{y}=\tau_{xy}=0$.

The stress state function z(x), satisfying all the above boundary conditions, is:

(18) $$z\left(x\right)=\left\{ \begin{array}{l} p,(\left|x\right|\leq a_{0})\\ \frac{px}{\sqrt{x^{2}-a_{0}^{2}}},(\left|x\right|>a_{0}) \end{array}\right.$$

where p is the frost heave force caused by water freezing. 

For convenience purposes, the origin of the coordinate system is shifted from the center of the crack to the right edge, and $r=x-a_{0}$ is the new coordinate. When $\left|x\right|>a_{0}$, Equation (18) can be rewritten as follows:

(19) $$z\left(r\right)=\frac{p\left(r+a_{0}\right)}{\sqrt{r^{2}+2ra_{0}}}$$

So, the stress intensity factor of the frost heaving force on the crack tip can be calculated as the following limit- form.

(20) $$K_{I}^{f}=\underset{\left|r\right|\to 0}{\lim}\sqrt{2\pi r}\cdot z\left(r\right)=\underset{\left|r\right|\to 0}{\lim}\frac{p\left(r+a_{0}\right)\sqrt{2\pi r}}{\sqrt{r\left(r+2a_{0}\right)}}=p\sqrt{\pi a_{0}}$$

### 4.4. Fracture Toughness 

**Figure 21:**
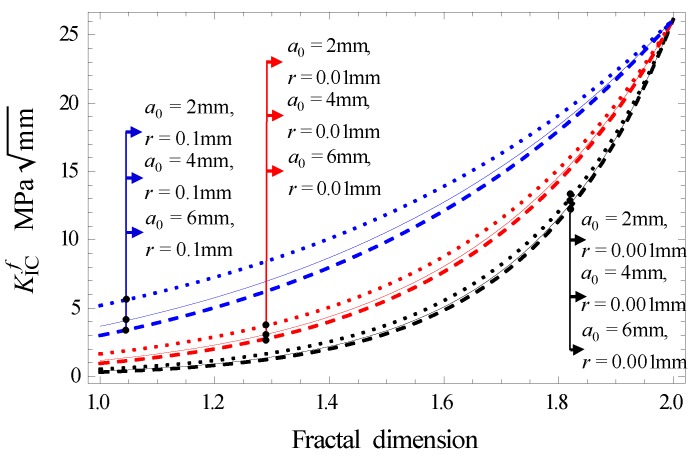
The relationship between fracture toughness and fractal dimension.

Based on the research of Wnuk [44] regarding the fact that stress fields at the crack tip as shown in Figure 21, the frost heaving stress $\sigma_{y}={\sigma_{\theta }|}_{\theta =0}$ at the tip of crack in *y*-direction can be calculated.

(21) $$\sigma_{y}=\frac{K_{I}^{f}}{{\left(2\pi r\right)}^{\alpha }}\left\{\cos\left(\alpha \theta \right)+\alpha \sin\theta \sin\left[\left(\alpha +1\right)\theta \right]\right\}$$

where $K_{I}^{f}$ is the stress intensity factor of frost heaving force, and α is the singularity order of the stress field for self-similar fractal crack. A noteworthy phenomenon in the theoretical studies of fractal fracture mechanics is the change of the order of stress singularity at the crack tip. For a fractal version of the Griffith crack, the familiar singularity of $r^{-1/2}$ is replaced by a weaker singularity for the near-tip stress $r^{-\alpha }$, where α depends on the fractal dimension $d_{f}$ [44].

(22) $$\alpha =\left\{ \begin{array}{l} 1-d_{f}/2,1\leq d_{f}\leq 2\\ 1.5-d_{f}/2,2<d_{f}\leq 3 \end{array}\right.$$

Once $\sigma_{y}=\sigma_{c}$, the stress intensity factor $K_{I}^{f}$ of frost heaving will reach its fracture toughness $K_{\mathrm{IC}}^{f}$. When an asphalt concrete crack expands, the expression of fracture toughness can be determined as follows:

(23) $$K_{\mathrm{Ic}}^{f}=\frac{2c{\left(2\pi r\right)}^{\alpha }\sqrt{d_{f}a_{0}^{d_{f}-2}\gamma E\delta^{3-\frac{2}{d_{f}}-d_{f}}}}{4^{\frac{1}{d_{f}}}\left\{\cos\left(\alpha \theta \right)+\alpha \sin\theta \sin\left[\left(\alpha +1\right)\theta \right]\right\}}$$

Seen from Figure 20b, a maximum cracking stress exists at *θ* = 0°. Equation (23), used to determine the maximum cracking stress ${\left(K_{\mathrm{Ic}}^{f}\right)}_{\max}$, can be simplified into Equation (24). The relationship between ${\left(K_{\mathrm{Ic}}^{f}\right)}_{\max}$ and fractal dimension of the different half crack length is shown in Figure 21.

(24) $${\left(K_{\mathrm{Ic}}^{f}\right)}_{\max}=2^{1-\frac{2}{d_{f}}}c{\left(2\pi r\right)}^{\alpha }\sqrt{d_{f}a_{0}^{d_{f}-2}\gamma E\delta^{3-\frac{2}{d_{f}}-d_{f}}}$$

As seen in Figure 21, the fracture toughness goes up with increments in the fractal dimension in the intervals 1–2. When the half-crack length is constant, the farther away from the crack tip, the greater the fracture toughness. This indicates that the distant part of the crack tip is less affected by the crack. When the distance from the crack tip remains constant, the larger the crack half-length, the smaller the fracture toughness. 

### 4.5. Certification

**Figure 22:**
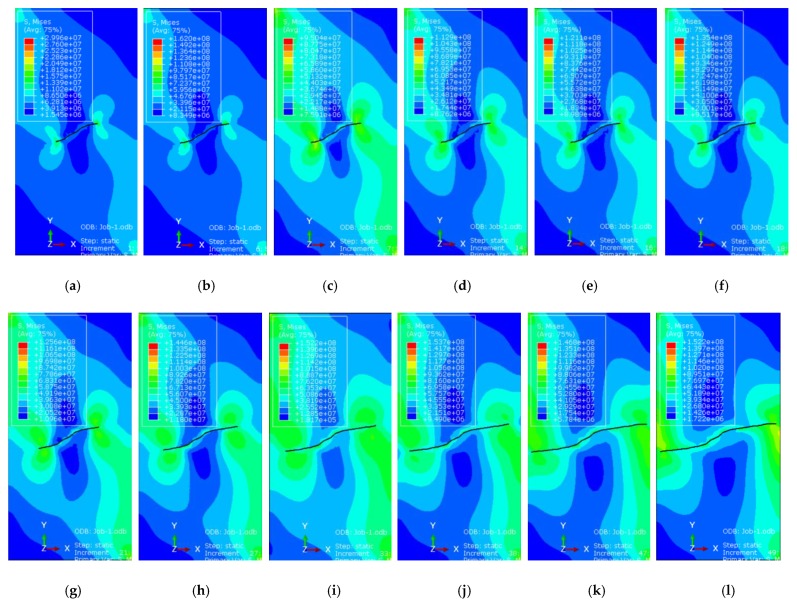
Dynamic evolution of fractal fractures in different time. (**a**) t = 0.01 s, (**b**) t = 0.05407s, (**c**) t = 0.5409 s, (**d**) t = 0.05526 s, (**e**) t = 0.05683 s, (**f**) t = 0.06034 s, (**g**) t = 0.07118 s, (**h**) t = 0.08267 s, (**i**) t = 0.08865 s, (**j**) t = 0.09205s, (**k**) t = 0.09370 s, (**l**) t = 0.09427 s.

In order to study the influence of freeze-thaw damage on fracture toughness, the simulation of fractal fractures after freeze-thaw cycling was carried out using ABAQUS. The extended finite element method (XFEM) is a new finite element method for solving fracture mechanics problems, which is widely used in the soil and rock fracture engineering field. The pictures in Figure 22 show the dynamic evolution of a prefabricated fracture with the fractal dimension (1.161). The spreading speed and geometry characteristic evolution with the time of freeze-thaw cycle = 0 were monitored [45].

As seen in Figure 22, the fractal fractures expand at the tip of crack over time, showing the characteristics of non-uniform propagation. When the stress intensity factor reaches the fracture toughness, the growth rate of the fracture will increase sharply, and the stress intensity factor can be used as the fracture toughness. 

**Figure 23:**
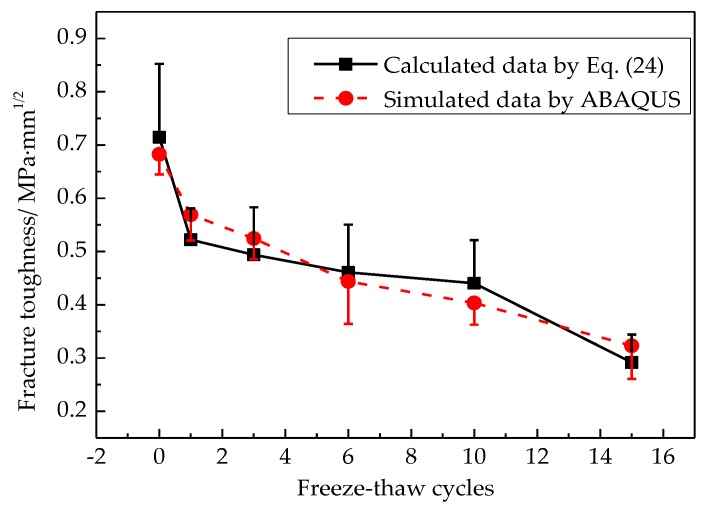
The comparison of calculated data and tested data.

The free surface energy density keeps constant in the process of freeze-thaw cycles, $\gamma =0.5{\;J/m}^{2}$, the fracture parameters and mechanical parameters are listed in Table 3. The comparison between calculation data from Equation (24) and the simulated data by using ABAQUS is shown in Figure 23.

As can be seen from Figure 23, fracture toughness decreases with an increase in the number of freeze-thaw cycles due to freeze-thaw damage. In other words, freeze-thaw cycles make asphalt concrete more brittle. The calculated data is in good agreement with the measured data, indicating that the fracture toughness calculation model, considering the fractal characteristics proposed in this study, is reasonable.

## 5. Conclusions

In this study, an image process for meso-damage fracture evolution in asphalt concrete was developed using the CT scanning technique. The pore and fracture were obtained nad analyzed by digital image processing techniques. Additionally, an evaluation of the mesoscopic fracture fractal behavior after freeze-thaw damage was undertaken, based on Griffith fracture theory. The model of critical crack stress, stress intensity factor, and fracture toughness with fractal characteristics were established. The conclusions may be summarized as follows:

1. A quantitative analysis of internal mesoscopic cracks could be regarded as the quantity index that reflected the freeze-thaw damage process of asphalt concrete. It confirmed that the fracture process of asphalt concrete accumulates gradually with the evolution of the cracks’ fractal dimensions. A transition develops from meso-crack to failure. Optical intensity was used to calculate the fractal dimension of the whole CT gray image, which ranged from 1.9 to 1.99.
2. The digital image processing technique was successfully applied to this study by introducing a series of software, such as MATLAB, IPP, MATHEMATICA, and MIMICS. The spatial distribution state of aggregates, asphalt mortars, and pores can be visualized using 3D reconstruction and threshold segmentation based on CT number. The fractal dimension of a single crack was obtained using the function of IPP measurement.
3. The law on the fracture and damage evolution is established by combining Griffith fracture theory and fractal theory. The calculation results show that critical stress grows with increasing the fractal dimension, and the longer the projection crack length, the less critical the crack stress will be. Meanwhile, the fracture toughness displays similar regularity. The calculation results, obtained using Equation (24), were very closed to the numerical simulation results obtained by ABAQUS.

## Figures and Tables

**Table 1:** Basic index for 90# petroleum asphalt.

Test Item		Unit	Test Result
Density (15 °C)		g/cm^3^	1.0364
Penetration (25 °C,100 g, 5 s)		0.1 mm	89
Softening point TR&B		°C	46
Flash point (COC)		°C	254
Solubility (solvent: trichloroethylene)		%	99.7
Wax content (distillation)		%	2.0
Ductility (15 °C, 5 cm/min)		cm	>150
Film oven heating test (163 °C, 5 h)	Mass loss	%	0.0198
	Penetration ratio	%	72.8
	Aging delay (25 °C)	cm	>150
	Aging delay (15 °C)	cm	>140

**Table 2:** Quality test results of the mineral powder.

Test Item		Unit	Test Result
Apparent density		g/cm^3^	2.73
Size range	<0.6 mm	%	100
	<0.15 mm	%	100
	<0.075 mm	%	89.5
Hydrophilic coefficient		-	0.87

**Table 3:** Fracture toughness and fractal dimension.

Freeze-Thaw Cycles	Specimen No.	*c*	$a_{0}$/mm	$d_{f}$	α	E/MPa	δ	$K_{IC}^{f}/\mathrm{MPa}\sqrt{\mathrm{mm}}$
0	S1	4.50	3.385	1.161	0.081	317.78	0.404	0.5324
	S2		1.235	1.155	0.078		0.307	0.8003
	S3		2.125	1.123	0.062		0.998	0.6695
	S4		1.648	1.203	0.102		0.155	0.6776
	S5		1.219	1.169	0.085		1.788	0.8932
1	S6	4.05	1.076	1.103	0.052	197.84	0.131	0.5859
	S7		1.582	1.120	0.060		1.109	0.5439
	S8		1.490	1.157	0.079		0.512	0.5406
	S9		1.761	1.195	0.098		0.040	0.4290
	S10		1.459	1.107	0.054		0.132	0.5108
3	S11	4.59	2.677	1.213	0.107	177.34	0.535	0.4641
	S12		1.985	1.156	0.078		0.187	0.4859
	S13		1.236	1.207	0.104		0.600	0.6342
	S14		1.685	1.122	0.061		0.066	0.4966
	S15		3.030	1.120	0.060		0.083	0.3880
6	S16	4.23	1.165	1.164	0.082	159.05	0.404	0.5545
	S17		1.327	1.174	0.087		0.307	0.5163
	S18		1.655	1.153	0.077		0.998	0.5025
	S19		2.531	1.176	0.088		0.155	0.3793
	S20		4.189	1.154	0.077		1.788	0.3512
10	S21	4.15	1.099	1.143	0.072	140.89	0.136	0.4951
	S22		2.855	1.112	0.056		0.020	0.3013
	S23		1.404	1.137	0.069		0.148	0.4484
	S24		1.187	1.185	0.093		0.326	0.4997
	S25		1.568	1.130	0.065		0.536	0.4572
15	S26	3.39	1.749	1.1772	0.089	133.25	0.299	0.3371
	S27		2.286	1.1479	0.074		0.044	0.2705
	S28		2.088	1.1944	0.097		0.081	0.2888
	S29		1.389	1.1372	0.069		0.072	0.3448
	S30		4.252	1.2023	0.101		0.081	0.2172
